# Genome-Wide Association Analysis and Candidate Gene Identification for Resistance to “Milky Disease” in the Chinese Mitten Crab (*Eriocheir sinensis*)

**DOI:** 10.3390/biology15030235

**Published:** 2026-01-27

**Authors:** Yilin Yu, Xiaochen Liang, Na Sun, Yan Zheng, Bingyu Li, Qingbiao Hu, Yingying Zhao, Yongan Bai, Xiaodong Li

**Affiliations:** 1 College of Animal Science and Veterinary Medicine, Shenyang Agricultural University, Shenyang 110866, China; yuyilin0722@163.com (Y.Y.); q1051079182@163.com (X.L.); huqingbiao@syau.edu.cn (Q.H.); zhaoyy@syau.edu.cn (Y.Z.); 2 Key Laboratory of Breeding and Reproductive Cultivation of Chinese Mitten Crab, Ministry of Agriculture and Rural Affairs, Panjin Guanghe Crab Industry Co., Ltd., Panjin 124200, China; sunna0911@163.com (N.S.); zhengyan2006126@126.com (Y.Z.); 13904278798@163.com (Y.B.); 3 College of Aquaculture and Life Sciences, Dalian Ocean University, Dalian 116023, China; libingyu@dlou.edu.cn

**Keywords:** *Metschnikowia bicuspidata*, milky disease, disease resistance, viable but non-culturable, molecular breeding

## Abstract

“Milky disease” of the Chinese mitten crab (*Eriocheir sinensis*), caused by *Metschnikowia bicuspidata*, leads to substantial economic losses. Despite extensive research on its pathogenesis, the genetic basis of host resistance and underlying regulatory mechanisms remain unclear, limiting the development of disease-resistant varieties. This study aimed to (1) evaluate resistance differences among 10 *E. sinensis* families (five highly resistant and five sensitive) via artificial challenge; (2) identify disease-resistance-associated genetic loci using genome-wide association analysis (GWAS). Our findings revealed that the F05 family exhibited the strongest resistance, with a mortality rate of only 3% and a molting rate of 73%, accompanied by superior growth performance. Whole-genome resequencing identified 10,161,545 high-quality SNPs, and GWAS detected 767 loci significantly associated with disease-resistance traits, among which two pleiotropic SNPs (Chr46:18395778 and Chr1:20680490) were simultaneously associated with both “dead or not” and “qPCR fixed amount of fungi”, reflecting their functional relevance in regulating survival and pathogen load. Notably, we propose for the first time that *E. sinensis* achieves resistance by inducing *M. bicuspidata* into a viable but nonculturable (VBNC) state, in which the fungal cells remain metabolically active but cannot form colonies on conventional culture media. This study establishes a multidimensional resistance evaluation system, clarifies the genetic basis and novel mechanism of resistance, and provides valuable molecular markers for marker-assisted breeding. The findings contribute to reducing disease-related losses and promoting sustainable development of the *E. sinensis* aquaculture industry.

## Simple Summary

The Chinese mitten crab (*Eriocheir sinensis*) is an important aquaculture species in China. However, “milky disease” caused by *Metschnikowia bicuspidata* poses a serious threat to crab health and leads to substantial mortality. To support the development of disease-resistant crab strains, we evaluated disease resistance across 10 crab families. The F05 family exhibited the highest resistance, with a post-infection mortality rate of only 3%, while maintaining normal molting and growth performance. Genome-wide analyses identified 767 loci significantly associated with disease resistance. Notably, our results suggest, for the first time, that resistant crabs may mitigate disease progression by inducing the pathogen into a viable but nonculturable (VBNC) state (metabolically active but non-culturable on conventional media). These findings provide a genetic and mechanistic foundation for selective breeding programs aimed at reducing disease-related losses and improving aquaculture efficiency.

## 1. Introduction

The Chinese mitten crab (*Eriocheir sinensis*) is one of the major freshwater aquaculture species in China, commonly referred to as the river crab or hairy crab [1]. In 2018, a novel disease characterized by hemolymph emulsification was first reported in farmed *E. sinensis* in the Panjin region of Liaoning Province [2] and was subsequently termed “milky disease.” Since then, the disease has spread rapidly across major crab-farming areas in Northern China, with infection rates exceeding 30% in overwintering ponds [3] and annual mortality frequently surpassing 20% [4]. These recurrent outbreaks have caused substantial economic losses and have become a major constraint on the sustainable development of the *E. sinensis* industry.

Studies have confirmed that the causative agent of milky disease is *Metschnikowia bicuspidata* [2]. During the early stage of infection, *M. bicuspidata* gradually colonizes tissues and organs such as muscle, hepatopancreas, and gills, while infected crabs typically do not exhibit obvious pathological symptoms. As infection progresses, diseased crabs exhibit reduced vitality, decreased appetite, opaque milky-white hemolymph and muscle, and the accumulation of milky fluid within the carapace cavity [2,5]. Further studies have shown that *M. bicuspidata* infection disrupts energy metabolism, growth and development, and immune-related metabolic pathways by altering the gut microbiota structure of *E. sinensis* [6], while exerting pathogenic effects through multiple synergistic pathways: On the one hand, it significantly suppresses the expression of key genes in the Toll signaling pathway, including *Toll1*, *Toll2*, *MyD88*, and *Dorsal*, thereby weakening innate immune capacity, impairing the antioxidant system, reducing phenoloxidase (PO) activity and antioxidant enzyme activities, and inducing severe oxidative stress characterized by elevated malondialdehyde (MDA) levels [5]. On the other hand, it disrupts gut microbiota composition, characterized by decreased abundance of Firmicutes and increased abundance of Proteobacteria, further exacerbating immune dysfunction in the host [7]. These combined effects severely suppress core immune functions (PO activity) and antioxidant capacity, while inducing pronounced energy metabolism disorders (extensive triglyceride consumption and elevated lactate dehydrogenase activity), ultimately leading to immune collapse and the death of the crab [5].

Following the first report of milky disease in 2018, subsequent studies have indicated that its occurrence is not solely determined by *M. bicuspidata* infection, but also influenced by multiple co-factors: environmental stressors can enhance host susceptibility by suppressing immune function [4] and alterations in the host gut microbiota can disrupt immune homeostasis, increasing vulnerability to *M. bicuspidata* [6]. These factors interact synergistically to affect the severity and prevalence of milky disease, highlighting the complexity of its etiology.

Genetic resistance to crustacean diseases follows two main models: polygenic resistance (regulated by multiple minor-effect loci) and oligogenic resistance (governed by a few major-effect genes). For example, resistance to white spot syndrome virus (WSSV) in Penaeus vannamei is typically polygenic [8], while resistance to bacterial cold water disease in rainbow trout is controlled by oligogenic loci [9]. In *E. sinensis*, previous studies have suggested that disease resistance may involve both adaptive immune components (e.g., Toll signaling pathway genes) and structural barriers (e.g., molting-related physical clearance) [10]. However, the relative contributions of these models to milky disease resistance remain unclear.

Despite the growing understanding of milky disease pathogenesis, effective control strategies remain limited, and the genetic loci underlying disease resistance have not yet been characterized. The widespread adoption of high-density farming practices has accelerated germplasm degradation in *E. sinensis*, manifested as reduced growth performance, increased environmental sensitivity, and heightened disease susceptibility [11]. With advances in technology, molecular breeding techniques have developed rapidly in recent years, and genome-wide association analysis (GWAS), which can identify genetic variants associated with complex traits or diseases, has broad application prospects for elucidating genetic mechanisms of complex diseases [12]. Wang et al. [11] reported that genetic breeding has improved growth performance but not disease resistance, indicating independent genetic control of these traits.

The viable but non-culturable (VBNC) state is a key reversible physiological adaptation of pathogens, in which cells retain metabolic activity and pathogenic potential but fail to form colonies on conventional media [13]. For crustacean aquaculture pathogens, existing studies only focus on environmental induction of the VBNC state: typical Vibrio pathogens of shrimp and marine crabs enter the VBNC state under low temperature or nutrient limitation and retain virulence after resuscitation [14], while *Vibrio vulnificus* VBNC cells show enhanced stress resistance, further confirming their latent threat to crustacean aquaculture [15]. Macey et al. [16] found that hemolymph of the blue crab (*Callinectes sapidus*) renders *Vibrio campbellii* non-culturable without elimination, yet this regulatory relationship has not been further explored. However, no studies have reported that *E. sinensis* resists milky disease by inducing the VBNC state to date, and its underlying genetic basis and regulatory mechanisms remain elusive.

In recent years, GWAS has become a key tool for dissecting the genetic basis of disease resistance in aquatic animals. Numerous GWAS studies have identified significant disease-resistance loci and candidate genes in different species, such as IRF8 and CD83 for rickettsial septicemia in Atlantic salmon [17]; CASP8 and TRAF3 for bacterial cold water disease in rainbow trout [9]; TLR7 and IL-8 for streptococcosis in tilapia [18]; apoptosis-related and immune-related genes as well as C-type lectin and Peritrophin for resistance to white spot syndrome virus in shrimp [8]; and C1q and Fibrinogen associated with oyster herpesvirus (OsHV-1) in Pacific oysters [19]. These studies collectively demonstrate that GWAS can effectively locate disease-resistance loci and identify candidate genes, providing a theoretical foundation for marker-assisted breeding and genomic selection.

The National Genetic Breeding Center for *E. sinensis* initiated breeding programs in 1999. After more than two decades of sustained efforts, a rich germplasm resource bank and over 200 dynamic families have been accumulated as research data and experimental materials. These abundant genetic resources provide strong support for conducting GWAS on milky disease resistance in *E. sinensis* and demonstrate the feasibility of this research. By applying GWAS to whole-genome scans of crab populations, it is expected to precisely locate gene loci associated with resistance to milky disease and elucidate the underlying genetic mechanisms. This will not only fill the research gap in gene mining for milky disease resistance in *E. sinensis* and provide a solid theoretical basis for resistance breeding but also facilitate the development of new crab varieties with high resistance, thereby promoting sustainable and healthy industry development.

## 2. Materials and Methods

### 2.1. Experimental Animals

All experimental *E. sinensis* were obtained from Panjin Guanghe Crab Industry Co., Ltd. (Panjin, China) Based on recapture rates and artificial challenge infection rates in the F1 generation of a disease-resistance breeding program, five families with high resistance to milky disease and five susceptible families were selected from a total of 189 families, resulting in ten experimental families. Population-level phenotypic data for these families are provided in Table A1. From each family, 15 females and 15 males with intact appendages and uniform size were selected, yielding a total of 300 experimental individuals. At the initial screening stage, no significant differences in growth-related traits were detected among the tested crabs, with an average body length of 21.33 ± 1.72 cm, body width of 23.50 ± 1.80 cm, body height of 10.80 ± 1.00 cm, and body weight of 5.80 ± 1.38 g.

### 2.2. Experimental Methods

#### 2.2.1. Artificial Challenge Experiment

The strain of *M. bicuspidata* used in this study was isolated from naturally infected *E. sinensis* in Panjin, Liaoning Province, identified by using PCR amplification and subsequent BLAST analysis (v2.17.0) of its ITS sequence on NCBI. The strain was activated on YPD medium at 25 °C for 48 h. For inoculum preparation, single colonies were inoculated into liquid YPD medium, shaken at 200 rpm, 25 °C for 24 h, and quantified using a hemocytometer.

**Figure 1:**
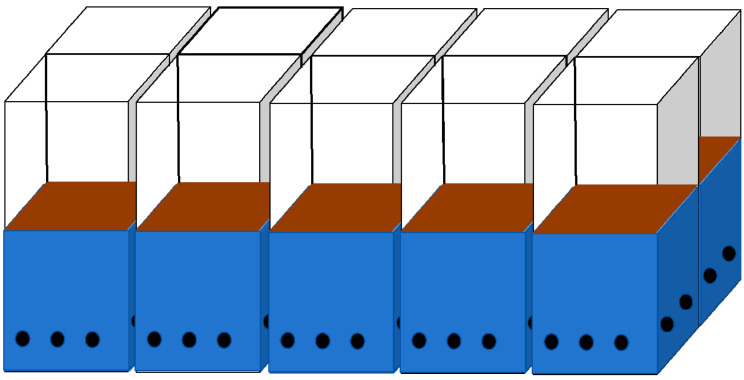
Schematic diagram of the individual rearing system (2 × 5 = 10 units per set, 30 × 10 = 300 units in total). Brown represents the mesh, black represents the drilled holes for water circulation, and blue represents the water surface.

Individual rearing units (20 cm × 20 cm × 50 cm) were used, each equipped with a mesh installed at the 20 cm water level to prevent escape and ensure complete immersion of the crabs. Ten individual units were assembled in a 2 × 5 arrangement, and holes were drilled at the bottom and sides to ensure water circulation. A total of 30 such assemblies were placed in an indoor concrete tank (10 m × 10 m × 10 m) to ensure that all crabs were maintained in the same water environment while being individually reared, thereby avoiding mutual interference (Figure 1).

The artificial challenge experiment was conducted for a duration of 60 days. Experimental groups (10 families, 30 individuals per family) were exposed to *M. bicuspidata* at 5 × 10^5^ CFU/L, while a negative control group (10 families, 30 individuals per family) was exposed to water without *M. bicuspidata*. All groups were maintained at 21 ± 1 °C, with 1/3 of the water replaced daily. Each group had three biological replicates (10 individuals per replicate).

#### 2.2.2. Sample Collection

During the experiment, each crab was inspected every two hours to record molting (phenotype “molt”) and mortality time (phenotype “dead or not” and “time of death”), and all morphological traits were measured again at the end of the experiment. Muscle tissue from the fourth walking leg of *E. sinensis* that died during the experiment and those euthanized at the end (via ice anesthesia: 10 min on crushed ice until locomotor activity markedly attenuated, followed by rapid dissection on ice). The sample collection and experimental procedures involving *E. sinensis* were approved by the Ethics Committee of Shenyang Agricultural University (Approval Code: 2022061402). All operations were conducted in accordance with the “Guidelines for the Ethical Treatment of Laboratory Animals in Liaoning Province” to minimize animal suffering.

All samples were stored in labeled PL8 aquatic sampling tubes (Shijiazhuang Boruide Biotechnology Co., Ltd., Shijiazhuang, China) for DNA extraction. Under sterile conditions, an appropriate amount of hepatopancreas tissue was collected and weighed for pathogen load determination using the culture method (phenotype “fungal culture fixed amount of fungi”), while another portion was stored at −20 °C for pathogen quantification by absolute fluorescence quantitative PCR (phenotype “qPCR fixed amount of fungi”).

### 2.3. Quantification of M. bicuspidata in Crab Hepatopancreas

#### 2.3.1. Fungal Culture Method

Hepatopancreas homogenization was performed using a tissue homogenizer (TissueLyser II, Qiagen, Hilden, Germany) at 50 Hz for 1 min, maintained on ice to avoid temperature elevation. The homogenization buffer was sterile physiological saline, with a tissue-to-buffer ratio of 1:10 (*w*/*v*). Then each sample was vortexed and subjected to serial dilution (10^1^–10^6^). An aliquot of 100 μL from each dilution was evenly spread onto Bengal red agar plates, with three replicates per dilution. Plates were incubated inverted for 48 h, after which colony numbers were counted.

#### 2.3.2. Absolute Quantitative Fluorescence Detection

The hepatopancreas from each crab was weighed, homogenized in 1000 μL of sterile water, vortexed, and subjected to serial dilution (10^1^–10^6^). An aliquot of 100 μL from each dilution was evenly spread onto Bengal red agar plates, with three replicates per dilution. Plates were incubated inverted for 48 h, after which colony numbers were counted.

PCR amplification of *M. bicuspidata* colony DNA was performed using primers HP-F (5′-AAACCCGCAAACTCCACAGA-3′) and HP-R (5′-TGGATATCACGCTCCATCATTT-3′). PCR products were purified using a Vazyme PCR product purification kit (Vazyme Biotech Co., Ltd., Jiangsu, China). Purified PCR products were ligated into the PMD™19-T Vector Cloning Kit using a reaction mixture containing 3 μL cDNA, 1 μL PMD19-T vector, 1 μL ddH_2_O, and 5 μL Solution I (total volume 10 μL). The ligation products were transformed into *Escherichia coli* DH5α competent cells and plated on LB agar, followed by overnight incubation at 37 °C. After confirmation of positive colonies, recombinant plasmids were serially diluted from 10^1^ to 10^9^ and used as templates. qPCR was performed according to the Vazyme kit instructions with the following program: initial denaturation at 95 °C for 3 min, followed by 40 cycles of 95 °C for 10 s and 63.5 °C for 30 s. A standard curve was generated by analyzing the relationship between CT values and serial dilutions of *M. bicuspidata*.

DNA of *M. bicuspidata* in the hepatopancreas of each crab was extracted using the BEasy™ Universal DNA Extraction Kit (Hangzhou BioEast Biotech. Co., Ltd. Hangzhou, Zhejiang, China). qPCR detection was performed following the method described by Liu et al. [20]. CT values were substituted into the standard curve equation to calculate *M. bicuspidata* load in the hepatopancreas of each crab (10^1^–10^9^ CFU/g).

### 2.4. DNA Extraction and Resequencing

Genomic DNA was extracted from muscle tissue of the last walking leg of each crab using the GenoPrep^®^ polysaccharide polyphenol kit (magnetic bead method; Shijiazhuang Boruide Biotechnology Co., Ltd., Shijiazhuang, Hebei, China).

DNA integrity was assessed by 0.8% agarose gel electrophoresis and quantified using Qubit 2.0 to ensure sufficient quality (≥150 ng per sample). Qualified DNA samples were used to construct resequencing libraries using the GenoBaits^®^ DNA Library Prep Kit for ILM. After library construction, preliminary quantification was performed using Qubit 2.0, followed by accurate quantification of effective concentration using qPCR to validate library quality. Qualified libraries were sequenced on the BGI MGI-2000/MGI-T7 platform using the PE150 mode (read length 150 bp), with an average sequencing depth of approximately 10× per sample. Raw reads were subjected to quality control to remove adapter sequences. Paired reads were discarded if more than 10 ambiguous bases (N) were present or if low-quality bases (Q ≤ 20) exceeded 40% of the read length. Clean reads were aligned to the reference genome using BWA with the mem algorithm to determine their genomic positions.

### 2.5. Analysis of Population Genetic Structure and Core Germplasm Selection

Based on the filtered high-quality SNP markers, which were obtained by excluding loci with MAF < 0.05, missing rate > 20% and non-biallelic variants, GCTA (v1.92.4) was first used to analyze kinship relationships to identify and exclude closely related individuals. Principal component analysis (PCA) was then conducted using Plink to explore patterns of genetic clustering among populations. To quantify population differentiation, multiple approaches were applied in this study. Genetic differentiation (F_ST_) among populations was calculated using Stacks. Population structure was analyzed using Admixture (v1.3), with the optimal K value determined based on cross-validation (CV) error to infer individual ancestry proportions. Pairwise linkage disequilibrium (r^2^) between SNPs was calculated using Haploview, and LD decay curves were generated. A phylogenetic tree was constructed using the neighbor-joining method in MEGA-X (model: p-distance; bootstrap: 1000 replicates). Finally, Core Hunter3 was applied to select and evaluate core germplasm resources using allele coverage as the evaluation criterion.

### 2.6. Genome-Wide Association Analysis

GWAS was performed using TASSEL (version: tassel-5-standalone-404d598d6b14) software for five phenotypic traits (“dead or not”, “time of death”, “fungal culture fixed amount of fungi”, “qPCR fixed amount of fungi”, and “molt”). The performance of four statistical models, including GLM, GLM (Q), MLM (K), and MLM (QK), was systematically compared. The MLM (QK) model was selected for GWAS of five phenotypic traits over GLM, GLM (Q), and MLM (K) due to its superior Q-Q plot fit and lowest false-positive rate. Q-Q plots were used to quantify model fit, with MLM(QK) showing the tightest alignment between observed and expected −log_10_(*p*)-values. Unlike other models, it exhibited minimal deviation from the null distribution for most SNPs, with only truly associated SNPs deviating beyond the confidence interval. The MLM (QK) model also achieved the lowest false-positive rate and was therefore selected for subsequent analyses. The optimal population structure inferred by Admixture (K = 14) was included as a fixed effect (Q matrix). The kinship matrix calculated by GCTA was incorporated as a random effect (K matrix). This was performed to control false positives caused by population stratification and relatedness among individuals. The significance threshold was determined using Bonferroni correction (*p* < 4.92 × 10^−9^). The Wald test *p* value for each SNP marker was used as the GWAS result. A threshold of *p* < 1.0 × 10^−5^ was used to define significant marker–trait associations.

### 2.7. Validation of Disease Resistance–Associated SNPs Identified by GWAS

A candidate SNP marker, LOC126981498, significantly associated with survival status and identified by GWAS, was selected, including three genotypes (GG, GT, and TT). Based on resequencing data, 292 crab DNA samples from 10 families were selected for validation. PCR amplification was performed using primers 56F (5′-ATGGACATCTTGTTCTTGCTGGG-3′) and 56R (5’-TCACTTCTTTATATCTCCTCTTATCCTCC-3′). The 50 µL PCR reaction mixture contained 5 µL DNA template, 2 µL 56F, 2 µL 56R, 25 µL 2× Rapid Taq Master Mix (Vazyme Biotech Co., Ltd.), and 16 µL ddH_2_O. PCR conditions were as follows: 95 °C for 3 min; 35 cycles of 95 °C for 15 s, 60 °C for 15 s, and 72 °C for 15 s; followed by 72 °C for 5 min. PCR products were examined by 1% agarose gel electrophoresis. PCR samples with clear bands and correct sizes were sent to Sangon Biotech (Shanghai) Co., Ltd. (Shanghai, China) for sequencing. Genotypes of each individual at this SNP locus were determined.

### 2.8. Gene Annotation

Candidate genes were functionally annotated using the online tool eggNOG-mapper (http://eggnog5.embl.de/#/app/guided_search, accessed on 10 January 2026). GO and KEGG enrichment analyses were conducted on annotated genes using the GO database, KEGG database, and the Omicshare online platform. The top 10 GO terms and top 15 KEGG pathways with the smallest *p* values were selected for visualization.

## 3. Results

A total of 300 experimental individuals (30 per family) were used. Multiple-testing correction was performed using Bonferroni correction (*p* < 4.92 × 10^−9^) to control false positive rates. Population structure and relatedness were addressed by incorporating the Q matrix (Admixture, K = 14) as a fixed effect and the K matrix (GCTA) as a random effect in the MLM (QK) model.

### 3.1. Phenotypic Data Results

#### 3.1.1. Growth Performance of Test Crabs

At the end of the experiment, the average growth per family was as follows: body length increased by 1.25 ± 2.31 cm, body width increased by 1.29 ± 2.26 cm, body height increased by 0.67 ± 1.08 cm, and body weight increased by 0.85 ± 0.96 g. Among them, the F05 family exhibited significantly superior growth performance compared with other families (*p* < 0.05), with body length increase of 2.03 ± 1.75 cm, body width increase of 2.15 ± 1.28 cm, body height increase of 1.15 ± 1.09 cm, and body weight increase of 1.61 ± 1.11 g.

#### 3.1.2. Molting and Mortality of Test Crabs

**Figure 2:**
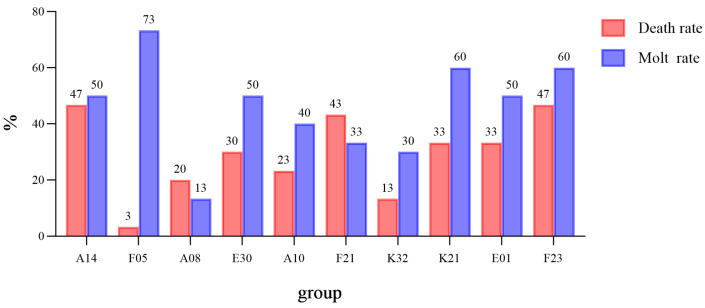
Death Rate and Molting Rate of Test Crabs in Each Family.

**Figure 3:**
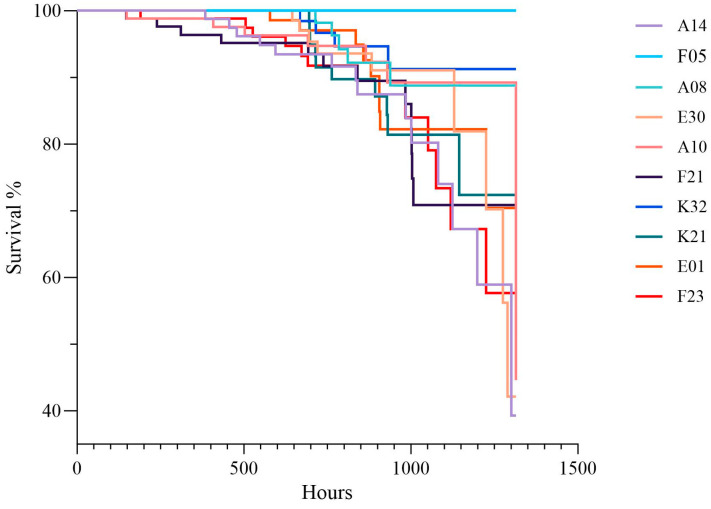
Survival Time of Test Crabs in Each Family.

During the experiment, crabs from all families underwent molting, with 46.9% of individuals completing molting. Regarding the relationship between mortality and molting rate (Figure 2), the F05 group exhibited the highest survival adaptability, with a molting rate of 73% and a mortality rate of only 3%, indicating a positive correlation between high molting rate and low mortality. Survival curve analysis (Figure 3) indicated that survival rates of all groups decreased over time, but the rates of decline differed significantly, with the F05 group showing the slowest decline.

#### 3.1.3. Load of *M. bicuspidata* in the Hepatopancreas of Experimental Crabs

**Figure 4:**
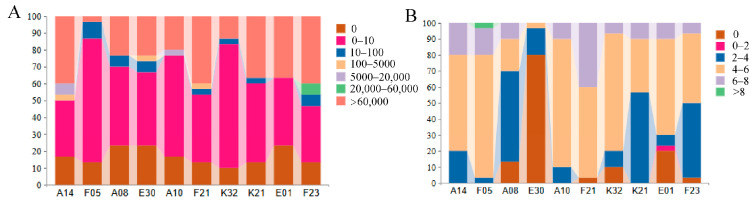
Load of *M. bicuspidata* in the hepatopancreas of test crabs in each family. Panel (**A**) shows the yeast load measured by fungal culture (CFU/g), and Panel (**B**) shows the yeast load measured by qPCR (10× scale).

The load of *M. bicuspidata* in the hepatopancreas of experimental crabs was simultaneously assessed using fungal culture methods (CFU/g) and qPCR (Figure 4), with results consistently indicating that: The total yeast gene copies detected by qPCR were significantly higher than the viable colony counts measured by culture, confirming that all family samples contained a substantial proportion of non-culturable yeast cells. Both methods revealed highly significant differences in yeast load among families: “fungal culture fixed amount of fungi” results showed that the F05 family had the fewest individuals with >60,000 CFU/g and the most individuals with 0–10 CFU/g; The viable yeast load in the F21 family was much higher than in other families, whereas E01 and K32 had the lowest loads; “qPCR fixed amount of fungi” results further confirmed this trend, with the F21, A14, and E30 families showing the highest total yeast loads. Notably, the culture-based load of the F05 family was not the highest, but its total load measured by qPCR ranked among the top, suggesting that this family has a particularly high proportion of non-culturable yeast.

The negative control group showed no clinical symptoms of milky disease during the 60-day experiment, with a mortality rate of 1% and undetectable *M. bicuspidata* in hepatopancreas (both culture and qPCR methods). This confirms that the observed phenotypes in experimental groups were specifically induced by *M. bicuspidata* infection, validating the reliability of phenotype assignment.

### 3.2. Whole-Genome Resequencing Results

A total of 45,528,072 SNPs were detected by whole-genome resequencing. After filtering, high-quality loci remained: 17,533,691 loci after removing those with minor allele frequency (MAF) < 0.05, 10,723,987 loci after removing loci with missing rates > 20%, and 10,161,545 loci after removing non-biallelic loci. Based on these filtered loci, samples were further filtered for completeness, retaining only those with SNP detection rates > 80%.

### 3.3. Population Genetic Structure Analysis

**Figure 5:**
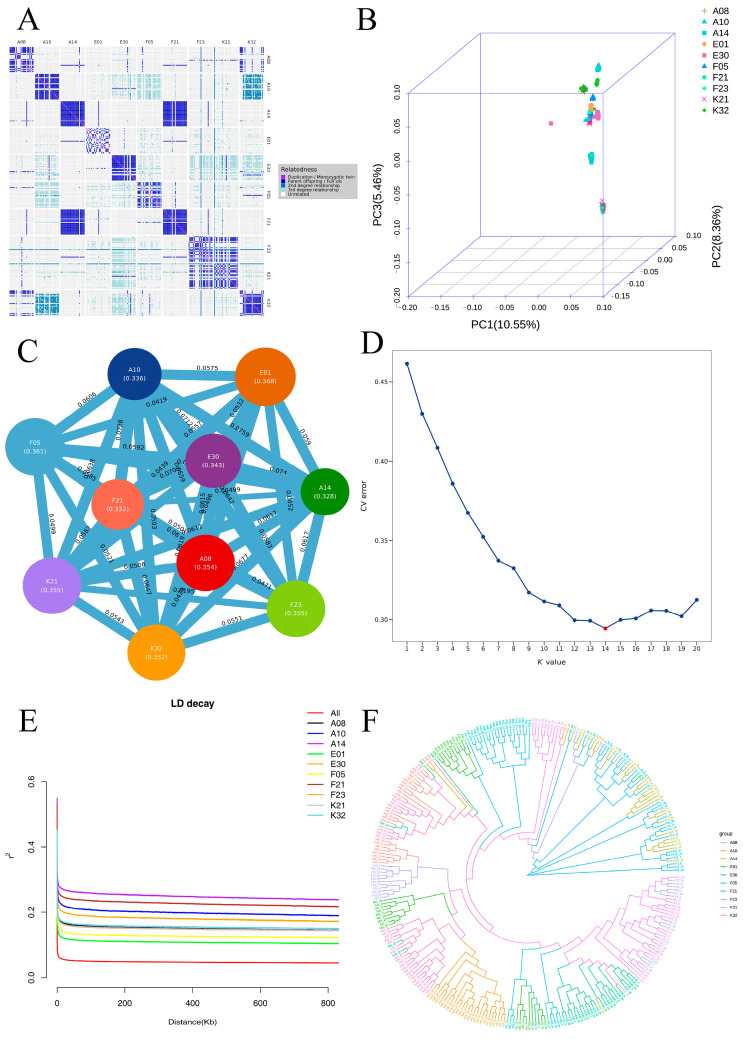
Population genetic structure analysis of 10 *E. sinensis* families ((**A**): Kinship heatmap; (**B**): PCA; (**C**): F_ST_ analysis; (**D**): Admixture ancestry composition; (**E**): LD decay curve; (**F**): Phylogenetic tree).

The kinship heatmap (Figure 5A) indicated generally weak genetic relatedness among samples, with most regions showing light coloration, though distinct clusters of closely related individuals were present within certain families. PCA based on the filtered SNPs (Figure 5B) revealed clear stratification in three-dimensional space: A14 and F21 families were distinctly separated along PC2, positioned in the negative range; A08, E01, E30, F05, F23, K21, and K32 families clustered relatively closely along PC2, with mean values ranging from 0.0161 to 0.0416. Along PC1, the A14 (0.0575) and F21 (0.0556) families were clearly separated from other groups. Along PC3, K21 (−0.0869) and F23 (−0.0755) families exhibited pronounced negative values, contrasting with the other groups.

Population differentiation F_ST_ analysis (Figure 5C) showed FST values ranging from 0.0049 to 0.0759. Some family pairs showed higher differentiation, with the highest F_ST_ observed between A14 and A10 (0.0759), followed by A14 vs. E30 (0.0739) and F21 vs. A10 (0.0728); K21 vs. F23 (0.0194) and A14 vs. F21 (0.0049) exhibited low differentiation. F_ST_ values of A14 relative to most families were generally high; F_ST_ between E01 and F05 was 0.0418, relatively low among combinations involving E01.

The optimal number of populations was determined as K = 14 based on cross-validation error rates, and the corresponding ancestry matrix effectively reflected sample ancestry (Figure 5D). Linkage disequilibrium (LD) analysis indicated that LD decayed rapidly with increasing physical distance (Figure 5E), with all populations showing similar decay patterns; the average LD decay distance (r^2^ declining to 0.1) was approximately 150–250 Kb.

Phylogenetic tree analysis (Figure 5F) revealed three major branches: one centered on A14 and F21 forming an independent evolutionary cluster, a second mainly comprising F23 and K21, and a third including the remaining samples (A08, A10, E01, etc.). The genetic distance between A14 and F21 was the shortest; individuals within each population were tightly clustered, and clear differentiation was observed between populations.

### 3.4. Results of Core Germplasm Analysis

**Figure 6:**
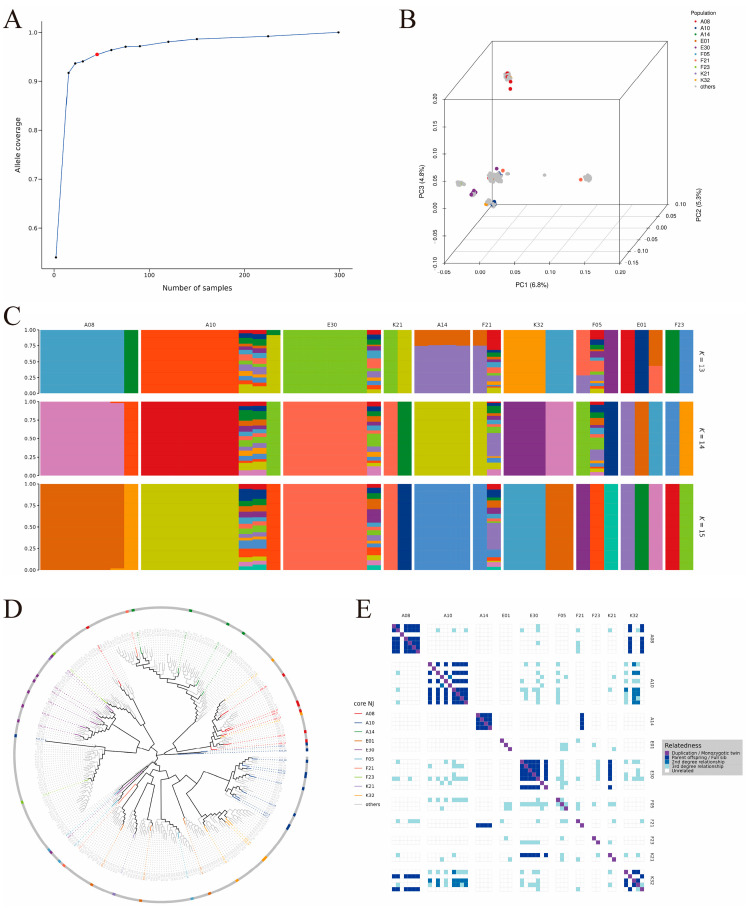
Core germplasm selection and validation analysis of *E. sinensis* ((**A**): determination of optimal core germplasm size; (**B**): PCA of core germplasm; (**C**): ancestry composition analysis of core germplasm; (**D**): phylogenetic tree of core germplasm; (**E**): kinship analysis of core germplasm).

In this study, the optimal size of the core germplasm was determined based on a 95% allelic coverage criterion (Figure 6A). Validation via principal component analysis (Figure 6B), population genetic structure (Figure 6C), and phylogenetic relationships (Figure 6D) confirmed that the core germplasm fully represents the genetic diversity of the original population. Genetic parameter evaluation (Table A2) indicated that the core germplasm maintains a high level of diversity, exhibits widespread heterozygote excess, and has a healthy genetic structure, with the K21 and F23 families performing particularly prominently. Kinship analysis (Figure 6E) further indicated that the core germplasm effectively removed many redundant individuals with close kinship in the original population. In the original population, individuals from families E30, F05, and A08 were overrepresented, whereas the core germplasm retained representative individuals from each family more evenly, significantly enhancing the uniqueness and representativeness of the resources. In summary, this core germplasm successfully achieved the goal of “maximizing genetic diversity while minimizing sample size.”

### 3.5. GWAS Results for Disease-Resistant Traits of E. sinensis

**Figure 7:**
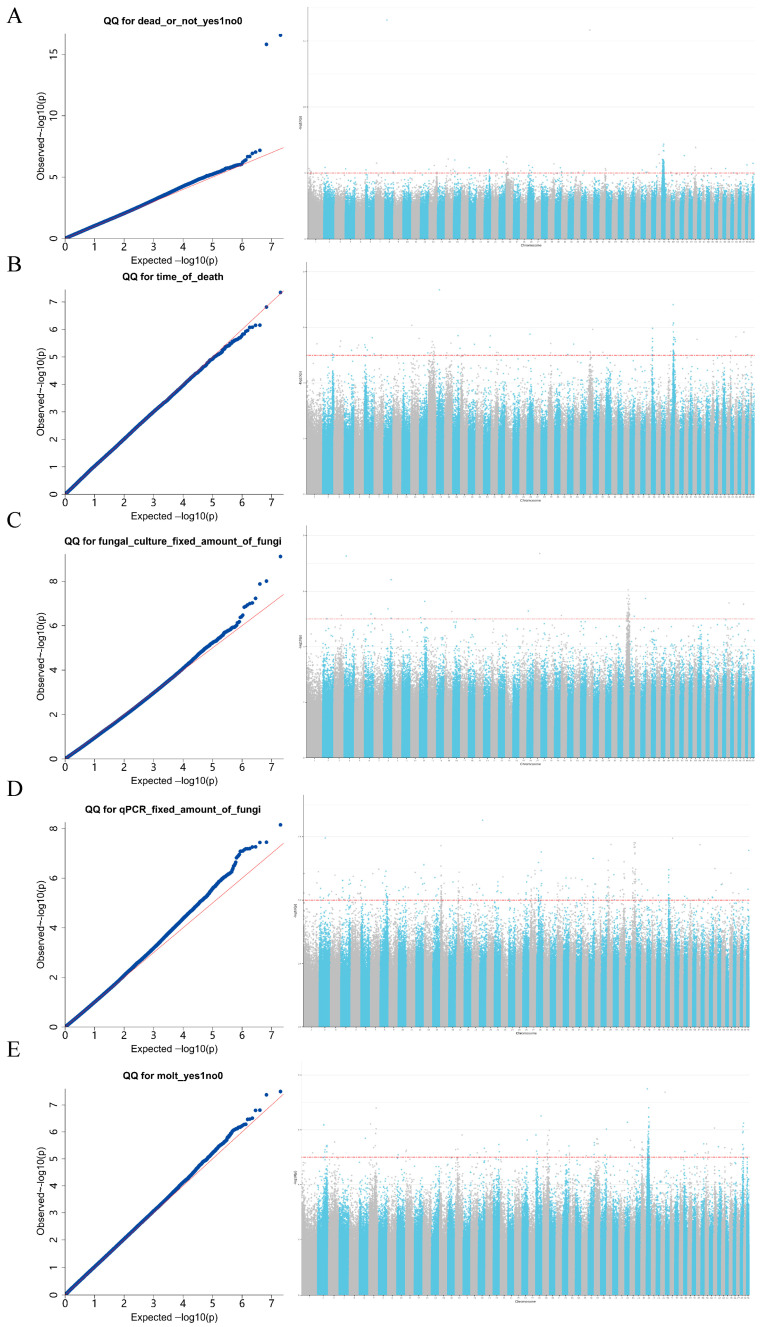
Results of GWAS for different phenotypic traits in *E. sinensis*. The left panels show quantile–quantile (Q–Q) plots, in which the y-axis represents the observed −log10(*p*) values and the x-axis represents the expected −log10(*p*) values; deviation of the curve from the diagonal indicates enrichment of association signals. The right panels display Manhattan plots, with −log10(*p*) values on the y-axis; the red dashed line indicates the genome-wide significance threshold (−lg*p* > 5), and peaks represent SNPs significantly associated with the corresponding traits. Phenotypes shown in each panel include (**A**): dead or not; (**B**): Time of death; (**C**): fungal culture with fixed amount of fungi (**D**): qPCR fixed amount of fungi; (**E**): molt. The red diagonal line represents the diagonal when the expected −log10(*p*) values are used for both the horizontal and vertical coordinates. The red dashed horizontal line represents the genome-wide significance threshold after Bonferroni correction.

The Manhattan and Q-Q plots of the genome-wide association analysis (Figure 7) showed that, based on the set genome-wide significance threshold (−lg*p* > 5), the following significant loci were identified: 189 loci associated with “dead or not”, 88 with “time of death”, 51 with “fungal culture fixed amount of fungi”, 287 with “qPCR fixed amount of fungi”, and 154 with “molt”. Among them, two SNPs (Chr46:18395778 and Chr1:20680490) were detected in both “dead or not” and “qPCR fixed amount of fungi”, indicating a pleiotropic effect. These two pleiotropic SNPs are located on chromosomes 46 and 1, respectively—chromosomes known to be enriched for immune-related genes (e.g., involved in stress response and pathogen recognition) in *E. sinensis* [21]. Their positions thus provide a direct genotypic link to the observed phenotypes of survival and pathogen load. After removing duplicate SNPs, a total of 767 significant loci were obtained. The Manhattan plot showed that SNPs associated with “dead or not” were mainly distributed on chromosome 48; SNPs associated with pathogen load by “fungal culture fixed amount of fungi” were mainly on chromosome 41; and SNPs associated with “molt” were primarily on chromosome 46.

### 3.6. Sequencing Results Validation

**Figure 8:**
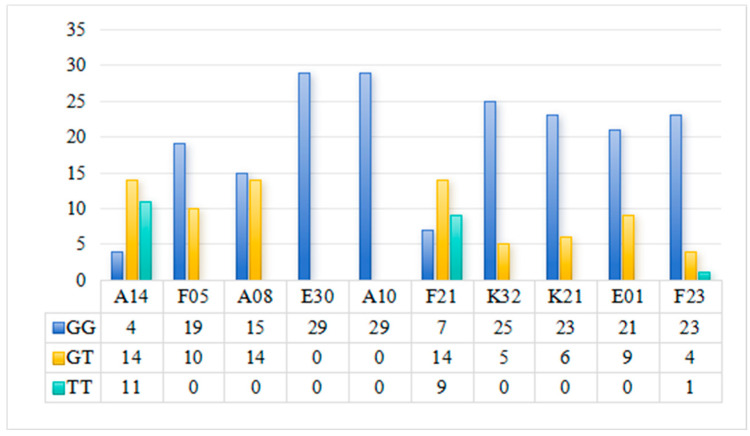
Detection results of mutation sites of marker LOC126981498 in 292 samples.

GWAS analysis revealed that the marker LOC126981498 was significantly associated with disease resistance. To verify the accuracy of the resequencing results, the three genotypes of this marker were first counted among 300 samples. Among all samples, the GG genotype accounted for 66.78%, GT for 26.03%, and TT for 7.19%. Sanger sequencing was performed on randomly selected samples, and the results (Figure 8) showed that among the 292 individuals with successfully obtained genotypes, the mutation status of marker LOC126981498 was consistent with the reported data, fully confirming the accuracy of the original GWAS genotype data. Further association analysis showed that the TT genotype had the highest death rate (57.14%), which was significantly higher than the GT genotype (32.89%) and GG genotype (27.69%); meanwhile, the culturable pathogen load in the hepatopancreas of TT genotype individuals (2.7 × 10^7^ CFU/g) was significantly higher than that of GT (3.73 × 10^6^ CFU/g) and GG (4.53 × 10^6^ CFU/g) genotypes, demonstrating that this SNP marker can effectively distinguish disease resistance levels and has practical utility for marker-assisted breeding.

### 3.7. Candidate Gene Identification and Functional Annotation

GO enrichment analysis was performed on differentially expressed genes associated with five milk disease resistance-related phenotypes, yielding 767 significantly enriched terms covering cellular components, molecular functions, and biological processes.

**Figure A1:**
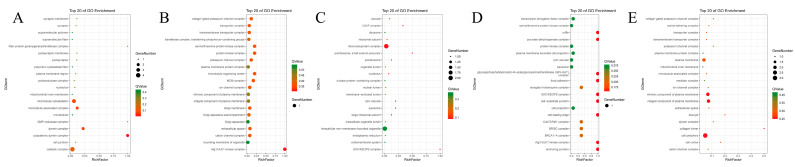
GO enrichment analysis of candidate genes related to disease resistance phenotypes within the Cellular Component. Phenotypes shown in each panel include (**A**): dead or not; (**B**): Time of death; (**C**): fungal culture with fixed amount of fungi (**D**): qPCR fixed amount of fungi; (**E**): molt.

In the biological process category (Figure A1), the “dead or not” phenotype was significantly enriched in steroid hormone-mediated signaling and response to steroid hormones; the “time of death” phenotype was enriched in vesicle organization and vesicle-Golgi fusion processes; the “fungal culture fixed amount of fungi” phenotype mainly involved tRNA threonylcarbamoyladenosine modification and metabolism; the “qPCR fixed amount of fungi” phenotype was enriched in tRNA pseudouridine synthesis and transcription elongation regulation; and the “molt” phenotype was closely associated with transmembrane receptor protein tyrosine kinase signaling and epithelial cell proliferation regulation.

**Figure A2:**
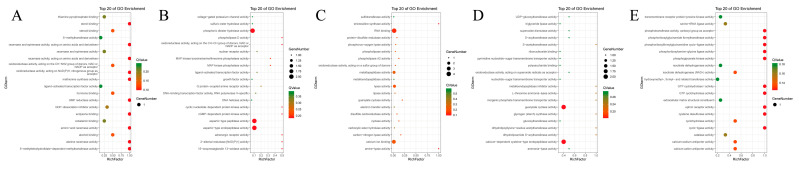
GO enrichment analysis of candidate genes associated with disease resistance phenotypes in Molecular Function. Phenotypes shown in each panel include (**A**): dead or not; (**B**): Time of death; (**C**): fungal culture with fixed amount of fungi (**D**): qPCR fixed amount of fungi; (**E**): molt.

Molecular function analysis (Figure A2) showed that the “dead or not” phenotype was mainly enriched in aspartic-type endopeptidase activity and glutathione–cysteine ligase activity; the “time of death” phenotype was enriched in phospholipase D activity and hydrolase activity related to membrane metabolism; the “fungal culture fixed amount of fungi” phenotype was enriched in guanylate cyclase and cyclase activities; the “qPCR fixed amount of fungi” phenotype was enriched in sequence-specific DNA binding and tRNA pseudouridine synthase activity; and the “molt” phenotype was enriched in transmembrane receptor protein tyrosine kinase activity and signal transduction adaptor activity.

**Figure A3:**
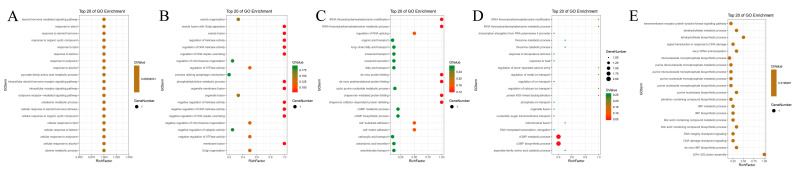
GO enrichment analysis of candidate genes associated with disease resistance phenotypes in Biological Process. Phenotypes shown in each panel include (**A**): dead or not; (**B**): Time of death; (**C**): fungal culture with fixed amount of fungi (**D**): qPCR fixed amount of fungi; (**E**): molt.

In the cellular component analysis (Figure A3), all five phenotypes were enriched in specific protein complexes. The “dead or not” and “time of death” phenotypes were co-enriched in the ATG1/ULK1 kinase complex and microtubule organizing center, relating to autophagy and cytoskeleton structures; the “fungal culture fixed amount of fungi” phenotype was specifically enriched in EKC/KEOPS complexes related to translation; the “qPCR fixed amount of fungi” phenotype was enriched in serine/threonine protein kinase complexes and cytoplasmic ribosomes; and the “molt” phenotype mainly localized to the cell cortex and cell–cell junctions.

Different phenotypes exhibited distinct enrichment patterns. Survival-related phenotypes (A, B) mainly involved stress response and cellular homeostasis regulation; pathogen load phenotypes (C, D) focused on gene expression and metabolic regulation; and the “molt” phenotype (E) was closely associated with developmental signaling pathways.

Comparing the two pathogen detection methods, the qPCR method revealed enrichment more focused on upstream gene expression regulation (e.g., transcription elongation, DNA binding), while traditional bacterial culture mainly reflected metabolic-level responses (e.g., tRNA modification, cyclase activity). qPCR detected more significantly enriched terms across all three GO ontologies (CC: 75, MF: 200, BP: 235), indicating that molecular detection methods have higher sensitivity in revealing host transcriptome responses.

KEGG enrichment analysis revealed the core biological processes underlying different traits and statistical models. Genes associated with survival-related phenotypes were significantly enriched in RNA degradation and ATP-dependent chromatin remodeling pathways, indicating that their genetic basis mainly involves fundamental regulation of gene expression. For pathogen load phenotypes, genes identified by the GLM model were highly enriched in complex cellular signaling pathways, including neurotrophic factors, mTOR, and insulin pathways, whereas the FarmCPU model showed significant enrichment in ribosome and proteasome pathways involved in basic genetic information processing, indicating that different statistical models capture genetic variations at different biological levels. Additionally, GWAS identified novel genes previously unreported in *E. sinensis* resistance to “milky disease,” whose enrichment analysis pointed to calcium signaling and cGMP-PKG pathways, suggesting that these genes may play important roles in cellular signal transduction.

## 4. Discussion

### 4.1. Family Resistance Differences and Phenotypic Associations

In this study, we established a comprehensive evaluation system integrating survival time, mortality, and pathogen load in the crab hepatopancreas through artificial infection experiments. Results revealed significant differences in “milky disease” resistance among the different families. Among them, the F05 family exhibited the highest survival rate and the lowest number of culturable pathogens in the hepatopancreas, indicating stronger disease resistance; this resistance advantage was positively correlated with growth traits, suggesting a possible shared genetic basis that warrants further exploration through genetic parameters and QTL mapping.

Importantly, the disease-resistant F05 family exhibited a unique pattern: high total pathogen DNA load detected by qPCR but low culturable colony counts, which directly supports the hypothesis that resistant crabs induce *M. bicuspidata* into a VBNC state. This suggests that the resistance mechanism may not solely involve preventing pathogen colonization or accelerating clearance, but rather inducing pathogens into a VBNC dormant state [16]. Such a phenomenon has been reported in crustaceans, for instance, in *C. sapidus* hemolymph, where *V. campbellii* is induced into VBNC, drastically reducing culturable bacteria while maintaining intact and metabolically active cells [16], which is consistent with the high DNA load yet low culturable counts observed in F05. Therefore, we hypothesize that the F05 family may, through enhanced innate immune responses, create a stressful microenvironment that forces invading pathogens into a metabolically dormant VBNC state. This “control” rather than “elimination” strategy offers a novel perspective for understanding the diversity of crustacean disease-resistance mechanisms: resistance may not only involve blocking or clearing pathogens but also suppressing them in a low-harm dormant state.

### 4.2. Population Genetic Structure and Core Germplasm Selection

The kinship heatmap revealed generally weak genetic relatedness among samples, indicating that the *E. sinensis* individuals selected in this study were largely independent and met the diversity requirements for population genetic analyses. In contrast, the presence of kinship clusters within specific groups suggested that some families may contain full-sibling or parent–offspring relationships, consistent with the commonly observed kin aggregation in crustacean populations [22]. These results were corroborated by phylogenetic tree and population structure analyses, providing critical evidence for subsequent data quality control and laying a foundation for selecting representative samples while minimizing bias from closely related individuals. Previous studies have shown that kin aggregation in natural populations of *E. sinensis* can bias genetic diversity estimates, necessitating stringent sample screening to ensure analytical accuracy [23].

Both PCA and phylogenetic tree analyses revealed that A14 and F21 possessed distinct genetic characteristics, with the shortest genetic distance observed between them. This suggests that they may share a common genetic origin or have experienced relatively recent gene flow. The pronounced differentiation between these two groups and the others may be associated with directional selection or differences in breeding strategies during artificial selection [11]. Similar patterns of population differentiation have been reported in cultured populations of *E. sinensis*. The primary driving forces underlying these patterns are often linked to restricted germplasm exchange or strong artificial selection pressure [24]. The distinct distribution of K21 and F23 along the PC3 axis, together with their formation of independent branches in the phylogenetic tree, further indicates pronounced genetic structural differences among *E. sinensis* families. This conclusion provides a theoretical basis for the classification, identification, and conservation of *E. sinensis* germplasm resources. It also offers valuable insights for addressing germplasm degradation issues observed in certain aquaculture regions [25]. Studies on related species, such as *Eriphia verrucosa*, have similarly demonstrated that geographic isolation and differences in reproductive strategies are key drivers of population genetic differentiation [26].

In this study, the overall F_ST_ values among *E. sinensis* families were low, indicating generally weak genetic differentiation among families. This result is consistent with the low genetic differentiation commonly observed in cultured populations of *E. sinensis*. This pattern may be attributed to frequent germplasm exchange and limited geographic isolation during aquaculture practices [27]. In contrast, some marine crab species exhibit similarly low inter-population F_ST_ values (0.08–0.16) due to their strong dispersal capabilities. This supports the role of dispersal and gene flow in shaping population differentiation [28]. Pfaller et al. [28] demonstrated in studies of pelagic drifting crabs that high levels of gene flow can substantially reduce genetic differentiation among populations. This observation is consistent with the genetic characteristics observed in the *E. sinensis* populations in this study. The high F_ST_ values between family A14 and most other families indicate that A14 possesses a distinct genetic background. This family can therefore be utilized as a valuable germplasm resource in breeding programs. In contrast, the low F_ST_ value between A14 and F21 confirms their high genetic similarity [24].

Through core germplasm selection, we successfully established a core collection that represents the maximum genetic diversity of the original population. This provides valuable material resources for long-term breeding programs and germplasm conservation. The widespread heterozygote excess observed in the core germplasm indicates that these families retain high levels of genetic diversity. This provides abundant genetic variation for the selection and improvement of disease resistance traits.

### 4.3. Genome-Wide Association Analysis Reveals the Genetic Basis of Disease Resistance

Using genome-wide association analysis (GWAS), this study identified many SNPs significantly associated with disease-resistance-related traits in *E. sinensis*. The chromosomal distribution patterns of these loci provide important insights. Loci associated with total pathogen load measured by qPCR were widely distributed across the genome. In contrast, loci associated with survival status and culturable viable pathogen load were predominantly enriched on chromosomes 48 and 41, respectively. This distributional divergence suggests that host survival and suppression of pathogen culturability may be governed by major-effect genes located in specific genomic regions. In contrast, the genetic mechanisms regulating total pathogen burden appear to be more dispersed across the genome [29]. This provides genetic evidence that “total pathogen load” and “viable pathogen load” are independent traits under distinct genetic regulation. This finding offers an intrinsic genetic explanation for the distinctive “high total load but low viable pathogen” phenotype observed in the F05 family.

Notably, two SNPs (Chr46:18395778 and Chr1:20680490) were simultaneously significant for both “dead or not” and “qPCR fixed amount of fungi”. This suggests that these SNPs may exhibit pleiotropic effects. Further validation is required through functional annotation of the candidate genes harboring these SNPs and analysis of the genetic correlation between the two traits. These results indicate that certain genes may ensure host survival by regulating pathogen “tolerance” or “control” mechanisms, allowing relatively high pathogen loads to persist. This mechanism closely aligns with previously reported phenomena in crustaceans in which host immune pressure induces pathogens to enter a viable but non-culturable (VBNC) state [30]. This may represent a key disease-resistance strategy in *E. sinensis* centered on reducing pathogen activity rather than eliminating pathogens entirely. In addition, loci associated with molting status were enriched on chromosome 46. This suggests that physiological clearance mechanisms play an important role in disease resistance [31]. Successful molting may serve as an effective physical defense mechanism. During molting, the immune system of *E. sinensis* undergoes remodeling, resulting in increased susceptibility to pathogens. Successful completion of molting reflects a favorable nutritional status and immune condition [10,32].

### 4.4. Functional Annotation of Candidate Genes and Dissection of Disease-Resistance Mechanisms

GO enrichment analysis was performed on differentially expressed genes associated with the five resistance-related phenotypes. This analysis revealed distinct yet complementary molecular mechanisms underlying these phenotypes. Survival-related phenotypes (“dead or not” and “time of death”) were significantly enriched in pathways related to steroid hormone response, autophagy (ATG1/ULK1 kinase complex), and proteolysis. Steroid hormone-mediated signaling pathways, enriched in survival-related traits, are known to regulate stress adaptation in crustaceans [29]—they may contribute to the stress-inducing internal microenvironment that drives *M. bicuspidata* into a VBNC state; meanwhile, autophagy pathways (ATG1/ULK1 kinase complex) can modulate pathogen survival status by regulating cellular homeostasis [28], collectively supporting the proposed VBNC-based resistance mechanism. This indicates that host survival outcomes are not solely determined by classical immune defenses. Instead, survival appears to be closely linked to hormone-mediated stress adaptation and maintenance of cellular homeostasis [33]. Steroid hormones play central roles in molting and stress responses in crustaceans. This suggests that successful resistance to infection may require integration of metabolic and developmental signals to reshape cellular states [34].

Notably, phenotypes corresponding to the two pathogen detection methods exhibited a clear hierarchical pattern. Genes associated with the culture-based method (reflecting culturable viable pathogens) were enriched in tRNA modification and guanylate cyclase activity. This points to alterations in host metabolic and translational environments required for active bacterial proliferation [35]. In contrast, genes associated with qPCR were significantly enriched in upstream functions such as transcriptional regulation and DNA binding. Moreover, the number of enriched terms was substantially higher than that observed for the culture-based method. At the molecular level, this confirms that qPCR more sensitively captures extensive transcriptional reprogramming of the host in response to pathogen presence. Such responses are more focused on global regulation of gene expression, rather than directly suppressing the metabolism of culturable pathogens.

The molting phenotype was uniquely enriched in receptor tyrosine kinase signaling and epithelial proliferation pathways. These highlights molting as a signal-intensive and resource-demanding developmental process. This process may involve resource competition or signaling crosstalk with anti-infective immune responses [36].

In summary, distinct dimensions of disease resistance are supported by differentiated molecular pathways. Survival depends on hormonal stress responses and cellular homeostasis. Control of pathogen load is associated with gene expression and metabolic regulation. Molting, in contrast, is linked to developmental signaling and tissue remodeling. These findings provide a multidimensional perspective for understanding the complex networks underlying disease resistance in crustaceans.

### 4.5. Limitations and Future Directions

This study has several limitations that need to be addressed in future research: (1) Functional validation of top candidate genes is lacking; subsequent studies will use RNA interference (RNAi) and overexpression techniques to verify their roles in milky disease resistance. (2) The GWAS results were based on a single population; replication in independent *E. sinensis* populations from different geographic regions is required to confirm the universality of identified SNPs.

Concrete future directions include (1) Integrating transcriptomics and epigenomics to link genetic variants to gene expression patterns during *M. bicuspidata* infection. (2) Conducting pilot marker-assisted selection (MAS) experiments using the identified SNPs (LOC126981498, Chr46:18395778, Chr1:20680490) to assess breeding efficiency.

## 5. Conclusions

By establishing a multidimensional evaluation framework incorporating survival time, molting status, and pathogen load, this study systematically revealed resistance differences to “milk disease” among different families of *E. sinensis*. The main conclusions are summarized as follows:

1. Significant differences in resistance exist among families. The F05 family exhibited the strongest overall disease resistance. Its high survival rate and molting success were significantly associated with low pathogen load. Moreover, its disease resistance advantage was consistent with superior growth performance. This suggests a potential shared or synergistic genetic basis between disease resistance and growth traits.

2. A novel disease-resistance mechanism was revealed. Resistant families exhibited a distinctive phenotype characterized by high total pathogen DNA load but low numbers of culturable viable pathogens. Combined with existing evidence, this study proposes for the first time that *E. sinensis* may achieve disease resistance by inducing pathogens into a viable but non-culturable (VBNC) dormant state. This provides a novel perspective for understanding the diversity of disease-resistance mechanisms in crustaceans.

3. The genetic basis and molecular regulatory networks of disease resistance were clarified. GWAS identified 767 SNP loci significantly associated with disease-resistance traits, including two pleiotropic SNPs (Chr46:18395778 and Chr1:20680490) co-regulating survival and pathogen load. Associated loci for different traits showed distinct chromosomal distributions, confirming that total pathogen load and viable pathogen load are independent traits under distinct genetic regulation. GO enrichment further revealed that survival traits rely on steroid hormone response/autophagy pathways, pathogen load traits rely on gene expression regulation, and molting traits rely on developmental signaling—each resistance dimension is supported by specific molecular pathways.

This study provides a systematic phenotypic evaluation framework, key molecular markers, and a novel mechanistic theory for disease-resistant breeding of *E. sinensis*. The identified 767 SNP loci, especially the functionally validated LOC126981498 and two pleiotropic SNPs, can be directly applied to disease-resistant molecular breeding of *E. sinensis* to accelerate the cultivation of high-resistance varieties and reduce industry losses caused by milky disease. These findings are significant for enhancing disease resistance and economic benefits in the *E. sinensis* aquaculture industry.

## Data Availability

The original data are provided in the article.

## Appendix A

**Table A1:** Basic Data of the Selective Breeding Families of *E. sinensis*.

Family	Infection Rate after Challenge/%	Recapture Rate/%	Average Size (Body Weight)/g	“Milky Disease” Resistance
A14	2.5%	26.4%	4.2	High Resistance
F05	12.5%	27.7%	3.6	High Resistance
A08	15.8%	30.3%	4.0	High Resistance
E30	17.5%	11.7%	8.6	High Resistance
A10	18.3%	17.8%	5.6	High Resistance
F21	70.0%	29.7%	4.5	Sensitive
K32	72.4%	17.2%	5.5	Sensitive
K21	75.0%	34.3%	4.7	Sensitive
E01	77.5%	30.6%	4.3	Sensitive
F23	89.7%	38.4%	4.8	Sensitive

**Table A2:** Genetic Parameter Evaluation of *E. sinensis* Families.

Family	Observed Heterozygosity (Obs Het)	Expected Heterozygosity (Exp Het)	Nucleotide Diversity (Pi)	Inbreeding Coefficient (Fis)
A14	0.47949	0.30355	0.34811	−0.23808
F05	0.46386	0.29832	0.40914	−0.08209
A08	0.46358	0.33474	0.40362	−0.1007
E30	0.47043	0.33653	0.37499	−0.17958
A10	0.45764	0.32451	0.35059	−0.19815
F21	0.46752	0.30962	0.41777	−0.07463
K32	0.47196	0.32877	0.35512	−0.22349
K21	0.45171	0.33113	0.39989	−0.0863
E01	0.46499	0.33446	0.35467	−0.21382
F23	0.46634	0.30753	0.41551	−0.07625
